# A retrospective study of elevated post-operative parathormone in primary hyperparathyroid patients

**DOI:** 10.18632/oncotarget.20416

**Published:** 2017-08-24

**Authors:** Shaobo Cao, Ya Hu, Yiming Zhao, Zhe Su, Zhiyan Xu, Xiang Gao, Quan Liao, Yupei Zhao

**Affiliations:** ^1^ Department of General Surgery, Peking Union Medical College Hospital, Chinese Academy of Medical Science & Peking Union Medical College, Beijing 100730, China

**Keywords:** primary hyperparathyroidism, normocalcemic parathormone elevation, parathyroidectomy, risk factors, recurrent hyperparathyroidism

## Abstract

We retrospectively analyzed the relationship between normocalcemic parathormone elevation (NPE) and recurrence of primary hyperparathyroidism (pHPT) after surgery, as well as the risk factors of NPE. Out of 309 patients with pHPT that underwent parathyroidectomy. Six months after surgery, 75 patients exhibited NPE with high preoperative serum levels of alkaline phosphatase, calcium and intact parathyroid hormone (iPTH), postoperative day 1 iPTH, and large parathyroid volume. 15 exhibited NPE at 2 years after surgery with low serum vitamin D levels. Postoperative serum iPTH levels gradually normalized in most patients. Multivariate analysis showed that male patients were at greater risk for postoperative NPE (p<0.05). Only 3 of 309 patients showed recurrence during the follow-up period. NPE may not predict recurrent hyperparathyroidism or incomplete parathyroidectomy for benign parathyroid lesions. Postoperative NPE thus appears to be a response to severe hyperparathyroidism and vitamin D deficiency.

## INTRODUCTION

Operation was the main treatment for primary hyperparathyroidism (pHPT) and the minimally invasive surgical approach had become increasingly popular recently [1]. After surgery, most patients are clinically cured demonstrating normocalcemia, normal serum parathyroid hormone and fewer disease symptoms. Nearly 12% to 45% patients show normocalcemic parathormone elevation (NPE) after successful parathyroidectomy [2–5]. However, the exact etiology of NPE is poorly understood [6]. It was reported that NPE after parathyroidectomy may imply incomplete parathyroidectomy or pHPT recurrence [7–9]. Approximately 3% to 7% of patients with persistent parathyroid hormone (PTH) elevation have recurrent pHPT [6]. Age, sex, preoperative serum calcium, PTH and serum vitamin D levels, body mass index (BMI), surgical approaches and pathology are probable risk factors for NPE [7–9]. Since postoperative NPE cannot be predicted, we conducted a retrospective study identify the risk factors that predict postoperative NPE and if it's related to recurrence of hyperparathyroidism.

## RESULTS

We followed up 334 patients with pHPT for more than 6 months and excluded 25 patients that didn't have the serum iPTH levels at the sixth month after parathyroidectomy. Therefore, 309 patients met the initial inclusion criteria and these included 65 male and 244 female patients. The mean follow-up time was 19.93±11.65 months and mean age was 53.58±12.92 years old. Among these, 6 patients were diagnosed with parathyroid carcinoma based on paraffin pathology.

### Analysis of clinical factors associated with NPE 6 months after surgery

Table 1 shows demographics and clinical data of patients with and without elevated serum iPTH at 6 months after surgery. At 6 months after surgery, 75 patients were diagnosed with NPE, whereas the remaining 234 patients had normal serum iPTH and calcium levels. In comparison to patients with normal iPTH at 6 months after surgery, the NPE patients had high pre-operative serum levels of alkaline phosphatase (347.47±446.63U/L*vs.* 150.79 ±216.34U/L, p=0.000), calcium (2.98±0.45mM *vs.* 2.85 ±0.33mM, p=0.000), iPTH (634.07±677.75pg/ml *vs.* 306.28±353.58pg/ml, *p*=0.000) and postoperative day 1 iPTH (49.95±78.10pg/ml *vs.* 28.59±40.91pg/ml, *p*=0.000) and demonstrated larger tumor diameter (24.44±11.05mm vs. 20.99±10.23mm, p=0.013). The men were at greater risk for NPE compared to women (p=0.005). In addition, the preoperative serum phosphate levels were lower in NPE patients (0.82±0.27mM vs. 0.84±0.18mM, *p*=0.037). However, the two groups of patients were similar in age, body mass index, surgical approach and preoperative serum levels of 25-hydroxyvitamin D and 1,25-dihydroxyvitamin D. In the multivariate analysis, preoperative serum levels of alkaline phosphatase, calcium, iPTH and phosphate, gender, pathology and recurrent pHPT were similar in patients with and without postoperative NPE.

**Table 1:** Comparison of demographics, clinical and biochemical data of primary HPT patients with normal and elevated serum iPTH levels 6 months after parathyroidectomy

Characteristics	Patients with NPE	Patients with normal iPTH levels	P
No. of patients	75	234	
Age (years)	50.80±14.00	54.47±12.46	0.146
Gender			0.005
Male	25	40	
Female	50	194	
Body mass index (kg/m2)	23.63±3.56	27.13±50.43	0.442
Preoperative serum 25OHD_3_ (ng/ml)^*^	12.93±7.79	14.67±15.39	0.325
Preoperative Serum 1,25(OH)_2_D_3_ (ng/ml)^**^	88.95±58.24	88.71±53.24	0.219
Preoperative serum alkaline phosphatase (U/L)	347.47±446.63	150.79±216.34	0.000
Preoperative serum phosphate (mM)	0.82±0.27	0.84±0.18	0.037
Preoperative serum calcium (mM)	2.98±0.45	2.85±0.33	0.000
Preoperative serum iPTH (pg/ml)	634.07±677.75	306.28±353.58	0.000
Gland weight (g)^***^	2.03±1.84	1.81±2.10	0.629
Gland diameter (mm)	24.44±11.05	20.99±10.23	0.013
Postoperative day 1 serum Ca (mmol/L)	2.26±0.46	2.31±0.21	0.980
Postoperative day 1 serum iPTH (pg/ml)^****^	49.95±78.10	28.59±40.91	0.000
Pathology			0.032
Carcinoma	4	2	
Others	71	232	
Recurrence pHPT	3	0	0.014
Follow-up (months)	18.58±12.43	20.35±11.38	0.202

25OHD_3_: 25-hydroxyvitamin D3; 1,25(OH)_2_D_3_: 1,25-dihydroxyvitamin D3;

^*^Data was available for 253 patients.

^**^Data was available for 225 patients.

^***^ Data was available for 204 patients

^****^Data was available for 259 patients.

### Characteristics of patients with recurrent pHPT

Among the 309 patients that underwent parathyroidectomy, three were diagnosed with recurrent pHPT. Also, postoperative paraffin pathology revealed that 2 patients had carcinoma (2/3) and 1 had adenoma. The two parathyroid carcinoma patients were diagnosed with hypercalcemia at 7 and 9 months after initial parathyroidectomy, respectively. Further surgeries did not cure hypercalcemia in these patients. The parathyroid adenoma patient was diagnosed with hypercalcemia at 16 months after surgery, and was kept under observation for 49 months without further surgery. Patients with normal serum iPTH levels at 6 months had no recurrence of pHPT during follow-up.

### Analysis of clinical factors associated with NPE 24 months after surgery

We followed up 136 patients for more than 24 months with an average follow-up of 31 months. Apart from the 3 recurrent cases, 15 patients showed NPE 24 months after surgery. These 15 patients received unilateral exploration or focused parathyroidectomy. Table 2 compares characteristics of patients with or without NPE at 24 months after surgery. We observed higher pre-operative serum levels of alkaline phosphatase (405.87±496.50U/L *vs* 183.32±299.97U/L, *p*=0.005), iPTH (720.64±731.31pg/ml *vs* 351.12±437.74pg/ml, *p*=0.001) and postoperative day 1 iPTH (71.01±108.94pg/ml *vs* 30.43±42.73pg/ml, *p*=0.014) in NPE patients at 24 months after operation than non-NPE patients. Preoperative serum 1,25(OH)_2_D_3_ (84.70±64.05ng/ml *vs* 91.78±42.08ng/ml, *p*=0.004) levels were lower in NPE patients at 24 months after surgery compared to non-NPE patients. Further, males were more at risk to have NPE at 24 months after surgery than females (*p*=0.000). However, both groups of patients were similar in age, BMI, gland weight, tumor diameter, and preoperative serum levels of 25OHD_3_, phosphate and calcium. In multivariate analysis, only male gender was independently associated with NPE at 24 months after surgery (odds ratio=10.767, 95% CI 1.52-20.49, *p*=0.004). None of the 136 patients had hypercalcemia during the follow-up.

**Table 2:** Comparison of demographics, clinical and biochemical data of primary HPT patients with normal and elevated serum iPTH levels 24 months after parathyroidectomy

Characteristics	Patients with NPE	Patients with normal iPTH levels	P
No. of patients	15	121	
Age (years)	50.2±9.81	53.0±12.80	0.261
Gender			0.003
Male	8	20	
Female	7	101	
Body mass index (kg/m2)	24.63±3.11	23.22±5.03	0.439
Preoperative serum 25OHD_3_ (ng/ml)^*^	11.24±5.75	13.45±8.83	0.892
Preoperative serum 1,25(OH)_2_D_3_ (ng/ml)^**^ (ng/ml)^**^	84.70±64.05	91.78±42.08	0.004
Preoperative serum alkaline phosphatase (U/L)	405.87±496.50	183.32±299.97	0.005
Preoperative serum phosphate (mM)	0. 79±0.20	0.82±0.17	0.318
Preoperative serum calcium (mM)	2.96±0.37	2.88±0.37	0.554
Preoperative serum iPTH (pg/ml)	720.64±731.31	351.12±437.74	0.002
Gland weight (g)^***^	2.15±2.54	2.48±4.16	0.758
Gland diameter (mm)	23.40±11.79	21.12±10.03	0.256
Postoperative day 1 serum Ca (mM)	2.40±0.25	2.29±0.17	0.054
Postoperative day 1 serum iPTH^****^ (pg/ml)^****^	71.01±108.94	30.43±42.73	0.001
Follow-up (months)	30.69±6.64	31.43±7.75	0.088

25OHD_3_: 25-hydroxyvitamin D3; 1,25(OH)_2_D_3_: 1,25-dihydroxyvitamin D3;

^*^Data was available for 108 patients.

^**^Data was available for 95 patients.

^***^ Data was available for 82 patients.

^****^Data was available for 107 patients.

### The percentage of patients with NPE at different follow-up times

**Figure 1:**
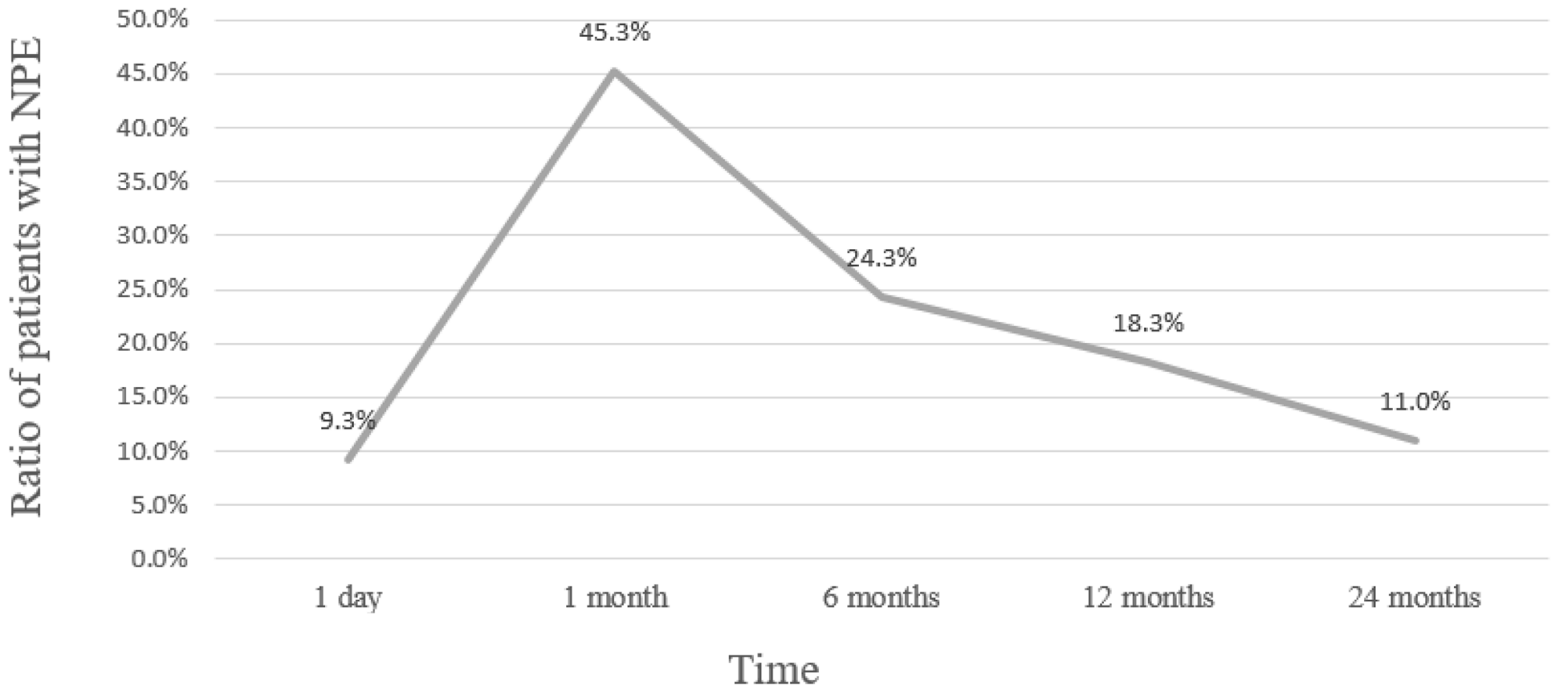
The percentage of patients with NPE at different follow-up times after parathyroidectomy

The data of postoperative day 1 serum iPTH was available for 259 patients, and 24 patients (9.3%) had elevated iPTH. The percentage of patients with NPE significantly raised from postoperative Day 1 to 1 month. Figure 1 shows that patients with postoperative NPE decreased from 45.3% at 1^st^ month to 24.3%, 18.3% and 11.0% at 6^th^, 12^th^ and 24^th^ month.

## DISCUSSION

Many patients with pHPT are diagnosed with NPE after successful parathyroidectomy. We observed that the incidence rate of NPE in our patients was 45.3%, 1 month after surgery. Although the overall postoperative iPTH levels normalized over time, 15 patients had elevated iPTH at 2 years after surgery and pHPT recurred in 3 patients. Long time follow-up of these patients is necessary to determine the etiology of NPE.

The risk factors for NPE are not clear. Previous studies have postulated vitamin D deficiency, bone hunger and impaired renal function as potential risk factors for NPE [7, 10–14]. Based on our results, we postulate that patients who had severe hyperparathyroidism with high preoperative serum calcium and iPTH levels are more prone to NPE post-operation. Also, NPE may be secondary to bone hunger and low vitamin D levels. As a common marker of bone disease and bone turnover, preoperative serum alkaline phosphatase levels were higher in patients with NPE at 6 and 24 months after surgery than those with normal serum iPTH. This suggested that patients with NPE had sever bone hunger before operation. Also, a more severe decrease from preoperative to postoperative day 1 serum calcium levels in NPE patients at 6 months after surgery suggested the presence of hungry bone syndrome. We observed low preoperative serum 1,25(OH)_2_D_3_ levels in patients with NPE, 24 months after surgery. This suggested that vitamin D deficiency was responsible for persistent PTH elevation in some patients. Vitamin D would decrease calcium absorption resulting in bone hunger and lead to excessive PTH secretion [15]. Patients with postoperative NPE had severe parathyroid disease with larger adenomas and high preoperative levels of serum calcium and iPTH. Our results were consistent to previous findings [3, 7, 10, 13, 16]. Beyer *et al* reported that patients routinely receiving oral calcium and vitamin D supplementation after parathyroidectomy reduced the incidence of NPE from 37% to 12% [10]. Mittendorf *et al* reported that 47% of the NPE patients had musculoskeletal symptom. They also demonstrated by multivariate analysis that musculoskeletal symptoms were associated with NPE risk and bone hunger played an important role in NPE. [16].

The clinical significance of NPE is still unknown. Much concern was focused on whether NPE represent recurrent pHPT, incomplete parathyroidectomy or operative failure. Previous studies reported that NPE was a risk factor for pHPT recurrence [7, 8, 17]. Solorzano *et al* showed that pHPT recurrence was more common in NPE patients than others [8]. However, many studies also showed that NPE was not associated with recurrence in their study group [3, 18–20]. They suggested that elevated PTH levels after surgery was an adaptation to bone hunger or vitamin D deficiency. But, these studies were limited by a relatively short follow-up time. In our study, 2 of the patients suffered pHPT recurrence were parathyroid carcinoma. Parathyroid carcinoma reportedly has a recurrence rate between 49% to 82% [21]. Other than the parathyroid carcinoma cases, both groups of patients were similar with respect to recurrent pHPT. None of the patients with normal serum iPTH 6 months after surgery showed recurrent pHPT. Operative failure has been defined as elevated serum calcium and PTH levels within 6 months after parathyroidectomy [3]. In our study, no patient suffered operative failure and NPE was not associated with incomplete parathyroidectomy. Also, Solorzano *et al* suggested that NPE did not indicate operative failure, but predicted recurrence in rare patients [8].

There were several limitations in our study. First, some biochemistry data were incomplete, which may have influenced the statistical analysis. Second, the follow-up was relatively short. Third, our data was a single center study, which may have resulted in selection bias.

In conclusion, our study showed that most patients with pHPT were cured after successful parathyroidectomy. However, a few patients demonstrated NPE postoperatively, but their numbers reduced over time. NPE may not indicate recurrent hyperparathyroidism for benign parathyroid lesions and elevated postoperative iPTH may be due to bone hunger and vitamin D deficiency. In future, a prospective study with a longer follow-up is necessary for pHPT patients after parathyroidectomy.

## MATERIALS AND METHODS

### Patient inclusion criteria

An electronic database was screened retrospectively for pHPT patients that underwent parathyroidectomy at Peking Union Medical College Hospital from June, 2012 to December, 2015. Patients were included if (1) they were diagnosed with pHPT by laboratory measurements, ultrasonography, technetium 99m sestamibi (MIBI) scan, and postoperative pathology; (2) followed up in our institution for more than 6 months; and (3) they were initially operated at our institution. The patients were excluded if (1) they were diagnosed with HPT-JT syndrome, familial or hereditary hyperparathyroidism or multiple neuroendocrine tumors; (2) they underwent parathyroidectomy at other centers; and (3) if they were followed up for less than 6 months.

### NPE characterization

NPE was defined as elevated intact PTH (iPTH) levels (>65 pg/ml) in combination with normocalcium levels (<2.72mM) at any time during follow-up period. Surgical cure was defined as normocalcemia more than 6 months after surgery. Recurrence was inferred if the patients maintained normocalcemia for at least 6 months after surgery followed by hypercalcemia later [4, 9, 18, 22, 23]. All patients were grouped according to the serum iPTH levels after parathyroidectomy. Promising predictors, such as age, gender, gland weight, tumor volume, preoperative and postoperative laboratory values were statistically analyzed.

### Parathyroidectomy and follow-up details

All patients underwent parathyroidectomy, localized by pre-operative MIBI scan and/or high-frequency ultrasonography. Focused parathyroidectomy with adequate preoperative imaging was available for the selective removal of a single parathyroid adenoma [24, 25]. All patients with a solitary adenoma underwent focused parathyroidectomy under cervical plexus anesthesia. Unilateral exploration (UNE) was undertaken if preoperative imaging indicated multiple abnormal glands on one side. Both focused parathyroidectomy and UNE are considered as minimally invasive parathyroidectomy (MIP). A bilateral exploration (BNE) was carried out under general anesthesia in patients with thyroid cancer, multi-nodular goiter and multiple abnormal glands on both sides. All the glands removed from the patients were verified by intra-operative frozen pathology. Postoperative paraffin pathology was conducted by two pathologists. All the patients received postoperative oral calcium and vitamin D supplementation for one month. Few patients received longer duration calcium and vitamin D supplementation based on symptoms and the biochemical data.

The following clinical data was collected: patient age and sex, BMI, maximum diameter of the tumor, preoperative serum levels of alkaline phosphatase, phosphate, 25-hydroxyvitaminD, 1,25-dihydroxyvitaminD, calcium and iPTH, and the postoperative pathology. Postoperative serum calcium and iPTH levels were also measured on day 1, 1^st^ month, 6^th^ month, 12^th^ months and 24^th^ month after surgery and annually thereafter.

### Statistical analysis

Statistical data was analyzed with SPSS software (version 16.0; SPSS Inc, Chicago, Illinois). A *p*<0.05 was considered statistically significant. Unless otherwise indicated, continuous variables were expressed as mean ± standard deviation (SD). For categorical data, statistical significance was analyzed by the chi-square and Fisher exact tests, whereas student's t test was used to compare continuous variables between groups. Multivariate regression analysis was also performed to identify the potential risk factors of NPE.
